# Motor difficulties and associated risk factors at 12 years in children born extremely preterm: a population-based cohort study

**DOI:** 10.1136/archdischild-2025-329687

**Published:** 2026-05-08

**Authors:** Maria Örtqvist, Nelly Padilla, Cecilia Montgomery, Karin Sävman, Thomas Abrahamsson, Aijaz Farooqi, Fredrik Serenius, Lisa B Thorell, Ulrika Ådén

**Affiliations:** 1 Department of Women's and Children’s Health, Karolinska Institutet, Stockholm, Sweden; 2 Medical Unit Allied Health Professionals, Karolinska University Hospital, Stockholm, Sweden; 3 Department of Women's and Children’s Health, Uppsala University, Uppsala, Sweden; 4 Department of Psychology, Uppsala University, Uppsala, Sweden; 5 Department of Pediatrics, Sahlgrenska Academy, University of Gothenburg, Gothenburg, Sweden; 6 Department of Neonatology, Sahlgrenska University Hospital, Gothenburg, Sweden; 7 Department of Biomedical and Clinical Sciences, Linköping University, Linköping, Sweden; 8 Crown Princess Victoria Children's Hospital, Linköping University Hospital, Linköping, Sweden; 9 Department of Clinical Sciences, Pediatrics, Umeå University, Umeå, Sweden; 10 Department of Clinical Neuroscience, Karolinska Institutet, Stockholm, Sweden; 11 Neonatal Unit, Karolinska University Hospital, Stockholm, Sweden

**Keywords:** Follow-Up Studies, Neonatology, Paediatrics, Rehabilitation, Adolescent Health

## Abstract

**Objective:**

To determine the prevalence of significant motor difficulties at 12 years of age in children born extremely preterm (EPT, <28 weeks’ gestational age) compared with term-born peers and to identify associated risk factors and comorbidities.

**Design:**

Population-based cohort study.

**Setting:**

National cohort from the Extremely Preterm Infants in Sweden Study, including children born from 2004 to 2007.

**Patients:**

Children born <27 weeks’ gestational age without cerebral palsy and/or severe neurodevelopmental impairment and term-born controls.

**Interventions:**

Motor function was assessed at 12 years using the Movement Assessment Battery for Children, second edition (MABC-2). Logistic regression identified risk factors for motor difficulties.

**Main outcome measures:**

Prevalence of significant motor difficulties, defined as scores ≤fifth percentile on MABC-2.

**Results:**

A total of 268 EPT-born and 196 term-born children were assessed. Significant motor difficulties were found in 30% of EPT-born children compared with 3% of term-born peers (adjusted OR 16.99; 95% CI 6.64 to 43.47). Male sex and lower gestational age were independently associated with higher risk. Among EPT-born children with motor difficulties, comorbidities were common, including autism spectrum disorders and attention deficit hyperactivity disorders. Only 9.5% of affected children had received physiotherapy in the previous year.

**Conclusions:**

Children born EPT, even in the absence of cerebral palsy and/or severe neurodevelopmental impairment, have a markedly increased risk of motor difficulties and associated neurodevelopmental comorbidities by age 12. Structured follow-up into mid-school age is essential to enable timely interventions.

**WHAT IS ALREADY KNOWN ON THIS TOPIC**

- Children born extremely preterm (EPT, <28 weeks’ gestation) are at high risk of motor impairments, but most studies focus on early childhood. Data on motor outcomes into mid-school age, particularly in those without severe neurodevelopmental impairments, remains limited.

**WHAT THIS STUDY ADDS**

- This study shows that 30% of children born EPT, even without cerebral palsy and/or severe impairments, have significant motor difficulties at 12 years of age—compared with only 3% of term-born peers. Male sex and lower gestational age are independent risk factors, and motor difficulties are frequently associated with neurodevelopmental comorbidities, such as autism spectrum and attention deficit hyperactivity disorders.

**HOW THIS STUDY MIGHT AFFECT RESEARCH, PRACTICE OR POLICY**

- Routine motor assessments should be extended into mid-school age for children born EPT, to support timely intervention and inform clinical guidelines and healthcare policies to better address long-term neurodevelopment in this population.

## Introduction

 There has been increased survival in children born extremely preterm (EPT; <28 weeks’ gestational age, GA) over the past decades but there is no trend in improved neurodevelopment.^1^ The prevalence of more severe motor difficulties, such as cerebral palsy (CP), has been relatively stable at around 11–12%, while an increasing trend in prevalence rates of non-CP motor difficulties, such as developmental coordination disorder (DCD) has been shown.^2^ Prevalence rates of non-CP motor difficulties reported in the EPT population greatly vary between 8 and >40%.^3–7^

Identifying motor difficulties in these children is essential from the earliest months of life and continuously, as these impairments can have effects that persist into school age.^8–13^ Motor skills are not only essential for everyday activities but also play a crucial role in the development of cognitive functions.^14 15^ Delays in motor skills can also limit participation in physical activities and social interactions, which in turn can impact mental health, academic progress and increase the risk for depression and anxiety,^11 16–19^ ultimately affecting the overall quality of life. This is supported by research from, for example, Husby *et al*,^20^ which highlights the long-term associations between motor skills and mental health, as well as health-related quality of life in young adults born preterm.^20^

The background of motor difficulties has been shown to be multifactorial, including both perinatal and neonatal risk factors.^7 21 22^ However, today, there is still a lack of studies examining motor outcomes and the predisposing factors beyond early childhood in EPT-born children.

There is evidence that various interventions have a positive effect on motor skills in children with non-CP motor difficulties such as DCD.^23–25^ However, the information is sparse regarding the use of motor-related healthcare in EPT-born children with non-CP motor difficulties. A large European study reported a high prevalence of non-CP motor difficulties at age 5, and most of these children were not receiving motor-related healthcare.^26^

The primary objective of the study was to evaluate the prevalence of significant non-CP motor difficulties in EPT-born children in Sweden at 12 years compared with healthy term-born controls. The secondary objectives were to analyse the relation between significant motor difficulties and perinatal, neonatal and sociodemographic risk factors and comorbidities in EPT-born children at 12 years, and to evaluate the parental view of the prevalence of motor difficulties and motor-related healthcare received.

## Methods

### Study population

Data from a prospective population-based study cohort, the EXPRESS (Extremely Preterm Infants in Sweden Study), including all children born EPT (<27 weeks GA) between 1 April 2004 and 31 March 2007, were included. In EXPRESS, children have been assessed at 2.5, 6.5 and 12 years. A control group was recruited initially for the 2.5-year follow-up^27^ and additional children were recruited at 6.5^28^ and at 12 years to substitute for attrition. Control children were selected from the Swedish Medical Birth Registry using the selection criteria, singleton birth, GA between 37 and 41 weeks, Apgar score at 5 min greater than 3. The control children were matched for zip-code, sex, day of birth and maternal country of origin. During all follow-ups, clinical examinations, parental questionnaires and standardised assessments evaluating cognition, neurology and motor skills were conducted. For this study, 12-year data of motor skills were used. The recruitment procedure, neonatal characteristics, neurodevelopmental outcomes at 1, 2.5 and 6.5 years have previously been reported.^27–29^ Baseline data, sociodemographic, prenatal, perinatal and neonatal characteristics in the EXPRESS study have been collected as previously described.^29^ Selection of risk factors for motor difficulties for this study was based on previous literature.^21^

Of the 486 surviving infants born EPT at 6.5 years, 462 (95%) were alive and eligible at 12 years of age, along with 373 children born at term (37–41 weeks GA). The predefined exclusion criteria of this study included CP (n=49) or other severe neurodevelopmental impairments (NDIs, n=36). Severe NDI was defined as having a severe cognitive impairment <3 SD (compared with the means and SD of the controls) on the fifth edition of the Wechsler Intelligence Scale for Children^30^ or severe hearing (deaf or remaining hearing problems despite hearing aids) or visual impairment (blind or visual acuity <20/400 of the better eye). 25 (51%) of the children with CP also had a severe NDI. One child in the term-born group was excluded due to severe cognitive impairment. Thus, a total of 377 EPT-born children and 372 term-born controls met the final inclusion criteria and comprised the eligible study population. To enable comparison with a previous study of the same cohort^3 21^ an additional analysis using a stricter exclusion criterion for cognitive function was performed—also excluding children with moderate cognitive deficits (<2 SD)—resulting in a subgroup of 192 children in the EPT group.

### Outcome measures

#### Movement assessment battery for children-second edition

At age 12, motor function was evaluated using the Movement Assessment Battery for Children-second edition (MABC-2), a widely used standardised tool with strong psychometric properties across multiple populations.^31^ The instrument comprises eight items across three subscales: manual dexterity, ball skills and balance skills. Raw test scores were converted into age-adjusted percentile scores, with subscale-specific and total standard scores calculated according to the manual.^31^ Motor function was classified as no motor difficulty (>15th percentile), at risk for motor difficulties (6th–15th percentile) or significant motor difficulties (≤5th percentile).^31^ Assessments were conducted by experienced paediatric physiotherapists at six university hospital follow-up clinics across Sweden, adhering to the standardised manual. As no Swedish norms exist, the original UK norms were applied. For this study, significant motor difficulties were defined as an overall MABC-2 score of ≤5th percentile.

### EXPRESS study-specific health questionnaire

Information on the parental view of the child’s overall gross and fine motor skills as well as the use of healthcare services, in regards to visits at a physiotherapist during the last year, was obtained from purposefully selected items of a study-specific parental questionnaire. The study-specific questionnaire was developed by the EXPRESS research group and was designed to capture descriptive information of parental perceptions about the functioning of the child, including a number of questions concerning gross and fine motor function and recent use of motor-related healthcare services (online supplemental eTable 1).

### Follow-up characteristics at 12 years

Information about autism spectrum disorder (ASD), attention deficit hyperactivity disorder (ADHD), language disorder and mild intellectual disability diagnoses was collected from the National Patient Register.^32–34^

### Statistical analysis

Continuous variables are presented as mean and SD, median with range or median with 25th–75th percentile where appropriate. Categorical variables are presented as frequency with percentage. For group comparisons, the Fisher’s exact test was used for dichotomous variables, Mantel-Haenszel χ² trend test for ordered categorical variables, χ² test for unordered categorical variables and Mann-Whitney U test or t-test for continuous variables depending on the variable distribution (checked by visual inspection of the histograms).

Adjusted logistic regressions were used for the main and secondary analyses to study the differences between EPT-born children and controls considering primary and secondary outcomes, adjusting for GA (in the EPT group), sex and multiple birth. Given the limited cluster sizes for families and the small number of assessment sites, multilevel models were not applied. As MABC-2 scores are age-standardised and the age range at follow-up was narrow, age at assessment was not included as a covariate in the regression models. Although MABC-2 scores were categorised into three groups for descriptive purposes, the regression analyses focused on a dichotomised outcome (≤5th percentile vs >5th percentile) to model clinically significant motor difficulties.

A sensitivity analysis for the prevalence of significant motor difficulties comparing children born EPT versus term-born controls, restricted to complete matched pairs with available MABC-2 total score was performed. This analysis was conducted because the original matching at 12 years of age could not be retained for all children in the cohort. This analysis included 126 complete matched pairs and was conducted using a conditional logistic regression model.

Sensitivity analyses regarding the prevalence of significant motor difficulties comparing children born EPT versus term-born controls, first excluding children with moderate and severe cognitive impairment and second, only including complete matched pairs (EPT-control) were performed.

The number of parameters in the model did not exceed the number of events/10. ORs with 95 CIs, and area under the receiver operating characteristic curve (AUC) was described.

The association analyses between patient characteristics and the primary outcome in the EPT population were evaluated using univariable logistic regression. Variables significant following Bonferroni-Holm adjustment from univariable analyses were then selected for further analyses in a multivariable model. A significant multivariable model was selected using stepwise forward and backward elimination selection. The variables sex, GA and multiple birth were forced into the model at all stages of the selection process.

All tests were two-tailed and conducted at 0.05 significance level following adjustment. All analyses were performed using SAS software V.9.4 (SAS Institute, Cary, North Carolina, USA).

## Results

### Study population

268 EPT-born children (boys n=139, weeks GA mean (SD) 25.0±1.0) and 196 children born at term (boys n=112, weeks GA mean (SD) 39.5±1.1) completed the motor assessment at 12 years (EPT group mean age 12.1±0.4 SD, control group 12.0±0.2) and were included in analyses, (figure 1). Descriptive data are presented in table 1. Significant differences (p<0.05) were found regarding both perinatal/neonatal and sociodemographic factors as well as comorbidities at 12 years between the two groups. Background characteristics, sociodemographic background and comorbidities at 12 years for EPT-born children with and without motor difficulties are reported in table 1. The EPT-born children with motor difficulties were more often boys, born at lower GA, with lower birth weights and had more neonatal morbidities (more days on mechanical ventilation, postnatal steroids, bronchopulmonary dysplasia (BPD) and severe retinopathy of prematurity), but had similar sociodemographic characteristics as the EPT-born children without motor difficulties. At 12 years, ASD and ADHD were also significantly more common in children born EPT with motor difficulties than without.

**Figure 1:**
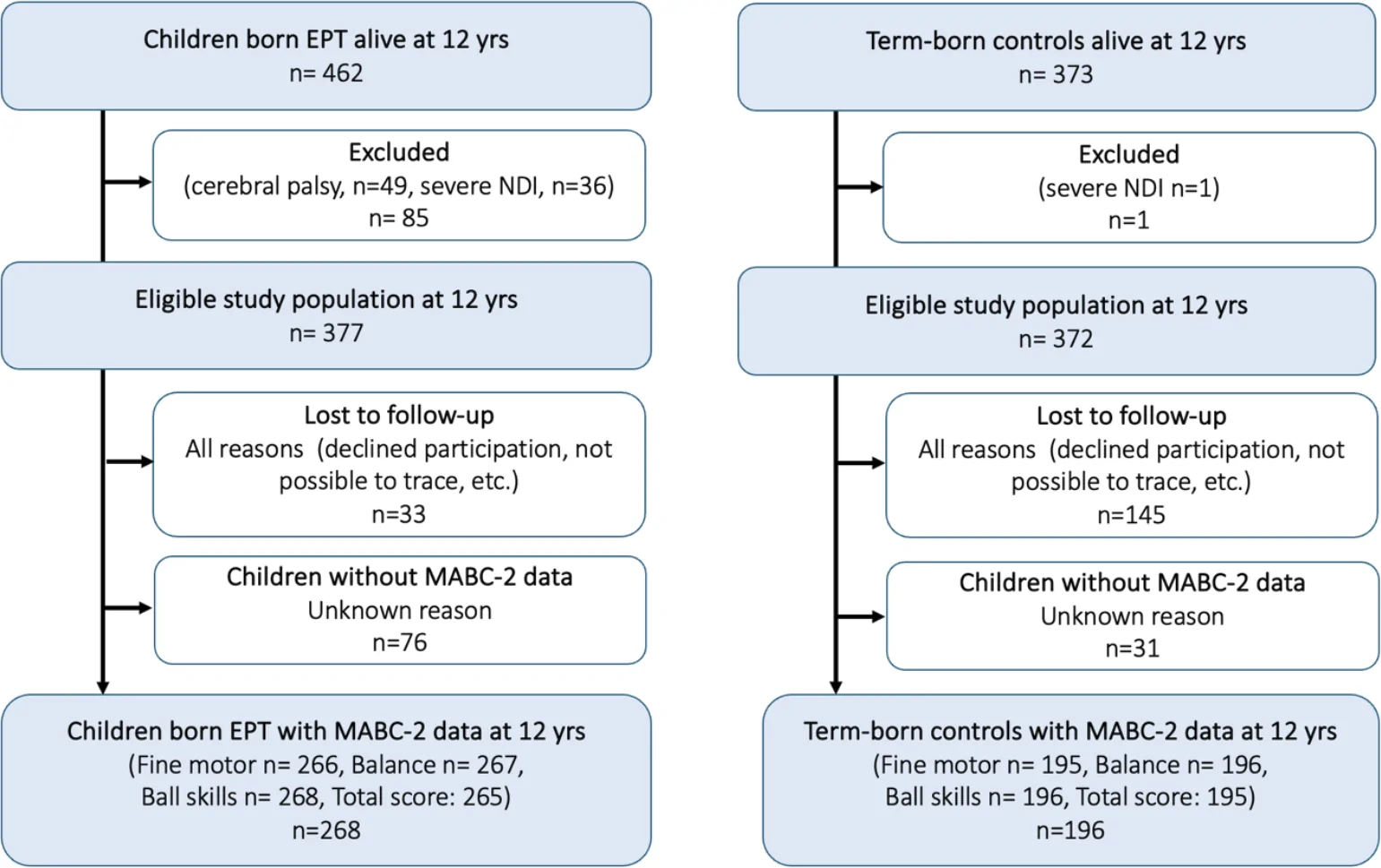
Flowchart of the study population. EPT, extremely preterm.

**Table 1:** Descriptive characteristics and comorbidity factors at 12 years for children born extremely preterm (without CP and/or other severe NDI) with and without significant motor difficulties and full-term born controls

Characteristics	Extremely preterm born All children n=268	Term-born controls All children n=196	P value*	Difference between groups* (95% CI)	Extremely preterm born		P value‡	Difference between groups‡ (95% CI)
					MABC-2 Total score ≤5th percentile n=79†	MABC-2 Total score >5th percentile n=186†		
*Perinatal and neonatal factors*								
Male sex, No. (%)	139 (52)	112 (57)	0.30	−5.3 (−14.4 to 3.9)	50 (63)	89 (48)	0.02	−15.4 (−28.3 to −2.6)
GA (weeks), mean±SD Median (range) IQR	25.0±1.0 25.0 (22.0–26.0) 24.0-26.0	39.5±1.1 40.0 (37.0–41.0) 39.0–40.0	<0.0001	−14.5 (−14.7 to −14.3)	24.5±1.0 24.0 (22.0–26.0)	25.2±0.9 25.0 (22.0–26.0)	<0.0001	0.7 (0.4 to 0.9)
Birth weight (g), mean±SD	792.9±164.5	3674.1±450.6	<0.0001	−2881.1 (−2947.6 to −2814.7)	716.3±145.1	824.4±162.4	<0.0001	108.1 (66.5 to 149.7)
Birth weight (z-score), mean±SD	−0.7±1.2	0.3±0.9	<0.0001	−1.0 (−1.2 to −0.8)	−0.9±1.3	−0.7±1.1	0.20	0.2 (−0.1; 0.5)
Small for GA§, No. (%)	44 (16)	1 (1)	<0.0001	15.9 (11.4 to 20.5)	16 (20)	28 (15)	0.37	−5.2 (−15.4 to 5.0)
Apgar score at 5 min <7, No. (%)	84 (31)	NA	NA		30 (38)	54 (29)	0.19	−8.9 (−21.5 to 3.6)
Pre-eclampsia, No. (%)	35 (14) n.d n=13	NA	NA		12 (16) n.d n=3	22 (13) n.d n=10	0.55	−3.3 (−12.8 to 6.3)
Primiparity, No. (%)	162 (60)	90 (46)	0.027	14.5 (5.4 to 23.6)	49 (62)	112 (60)	0.68	−1.8 (−14.6 to 11.0)
Multiple birth, No. (%)	52 (19) n.d n=1	0 (0)	<0.0001	19.5 (14.7 to 24.2)	16 (20)	36 (19) n.d n=1	0.92	−0.8 (−11.3 to 9.7)
Antenatal steroids (incomplete and complete), No.(%)	243 (91) n.d n=10	NA	NA		75 (96) n.d. n=1	165 (93) n.d. n=9	0.14	−2.9 (−8.6 to 2.7)
Postnatal steroids, No. (%)	58 (22)	NA	NA		24 (30)	33 (18)	0.03	−12.6 (−24.2 to −1.1)
Days on mechanical ventilation, Median (range)	7.0 (0.0–121.0)	NA	NA		14.0 (0.0–59.0)	6.0 (0.0–67.0)	<0.0001	−6.0 (−9.0 to −3.0)¶
0–7 days, No. (%)	136 (51)	0	NA		27 (34)	108 (58)	<0.0001	23.9 (11.3 to 36.5)
8–28 days, No. (%)	98 (37)	0	NA		33 (42)	64 (34)		−7.4 (−20.2 to −5.5)
>28 days, No. (%)	34 (13)	0	NA		19 (24)	14 (8)		−16.5 (−26.7 to −6.4)
Caesarean section, No. (%)	156 (58)	NA	NA		44 (56)	109 (59)	0.72	2.9 (−10.1 to 15.9)
Intraventricular haemorrhage, grade 3–4, No. (%)	17 (6) n.d n=1	NA	NA		6 (8)	10 (5) n.d n=1	0.57	−2.2 (−8.9 to 4.5)
Retinopathy of prematurity, stage III–IV, No. (%)	77 (29) n.d n=2	NA	NA		31 (40) n.d n=1	45 (24) n.d n=1	0.02	−15.4 (−27.9 to −2.9)
Periventricular leukomalacia, No. (%)	3 (1)	NA	NA		1 (1)	2 (1)	1.00	−0.2 (−3.1 to 2.7)
Necrotising enterocolitis, surgically treated, No. (%)	15 (6)	NA	NA		6 (8)	8 (4)	0.37	−3.3 (−9.8 to 3.2)
Bronchopulmonary dysplasia**, No. (%)	155 (64) n.d n=24	NA	NA		51 (74) n.d n=10	102 (59) n.d n=14	0.04	−14.6 (−27.3 to −1.9)
Patent ductus arteriosus, ligated, No. (%)	61 (23)	NA	NA		23 (29)	37 (20)	0.11	−9.2 (−20.8 to 2.3)
Sepsis, culture verified, No. (%)	125 (47)	NA	NA		41 (52)	82 (44)	0.28	−7.8 (−20.9 to 5.3)
*Sociodemographic factors*								
Mother’s age at infant delivery (years), mean±SD	31.2±5.8	31.2±4.8	0.93	−0.04 (−1.0 to 0.9)	31.8±5.8	30.9±5.8	0.22	1.0 (−2.5 to 0.6)
Maternal educational level, university/college, No. (%)	141 (53)	140 (71)	<0.0001	−18.8 (−27.5 to −10.1)	38 (48)	101 (54)	0.42	6.2 (−6.9 to 19.3)
Maternal country of birth, non-nordic, No. (%)	44 (17) n.d n=4	5 (3)	<0.0001	14.1 (9.1 to 19.1)	16 (21) n.d n=2	27 (15) n.d n=2	0.27	−6.1 (−16.5 to 4.3)
*Follow-up characteristics at 12 years*								
Age at assessment (years), mean±SD median (range)	12.1±0.4 12.0 (10.5–14.4) n.d n=12	12.0±0.2 11.9 (11.6–12.8) n.d n=6	<0.001	0.15 (0.09 to 0.21)	12.1±0.4 12.1 (10.5–13.9) n.d n=1	12.1±0.4 12.0 (11.7–14.4) n.d n=10	0.34	0.1 (−0.1 to 0.2)
Autism spectrum disorder, No. (%)	29 (11)	2 (1)	<0.0001	9.8 (5.8 to 13.8)	18 (23)	10 (5)	<0.0001	−17.4 (−27.2 to −7.6)
Attention deficit hyperactivity disorder, No. (%)	55 (21)	16 (8)	0.0002	12.4 (6.2 to 18.5)	29 (37)	25 (13)	<0.0001	−23.3 (−35.0 to −11.6)
Language disorder, No. (%)	19 (7)	1 (1)	0.0003	6.6 (3.3 to 9.8)	9 (11)	10 (5)	0.12	−6.0 (−13.7 to 1.7)
Intellectual disability (mild), No. (%)	6 (2)	0 (0)	0.042	2.2 (0.5 to 4.0)	4 (5)	2 (1)	0.07	−4.0 (−9.0 to 1.1)

For test between two groups with respect to dichotomous variables Fisher’s exact test was used, for ordered categorical variables Mantel-Haenszel Chi-square trend test, Chisquare test for unordered categorical variables, and for continuous variables Mann-Whitney U test or two-sample t-test.

For categorical variables the difference between groups were calculated as difference in proportion with corresponding 95% CI.

For continuous normally distributed variables, the mean difference between groups is presented with corresponding 95% CI.

* P value and 95% CI, comparison between all children born EPT and all children born at term.

† Children born EPT with a MABC-2 total score n=265 (missing n=3)

‡ P value and 95% CI, comparison between children born EPT with and without significant motor difficulties

§ Small for gestational age, birth weight <- 2SD below the mean of the Swedish standard for intrauterine growth.

¶ For continuous non-normally distributed variables, Hodges-Lehmann estimator is presented, corresponding to the median of all pairwise differences in the outcome between the groups, along with 95% confidence intervals.

** Bronchopulmonary dysplasia, supplementary oxygen at 36 weeks of gestational age.

CP, cerebral palsy; GA, gestational age; MABC-2, Movement Assessment Battery for Children-second edition; NA, not assessed; n.d., no data; NDI, neurodevelopmental impairment.

Neonatal and sociodemographic characteristics were largely comparable between EPT-born children included in the MABC-2 analyses and those without available MABC-2 data. However, the dropout group had a significantly higher proportion of non-Nordic mothers and a greater prevalence of periventricular leukomalacia (PVL) (online supplemental eTable 2). In the term-born group, the dropouts had a significantly lower birth weight and a higher proportion of non-Nordic mothers compared with the included children born at term (online supplemental eTable 3).

### Prevalence of significant motor difficulties

Children born EPT without CP and/or other severe NDI showed a significantly higher prevalence of overall motor difficulties (79/265, 30%) than children born at term (5/195, 3%) at the age of 12; OR adjusted for sex and multiple birth (aOR) 16.99, 95% CI 6.64 to 43.47, p<0.0001 (table 2). Significant group differences were also found for all subscales. The highest risk was seen for balance skills (aOR 26.38, 95% CI 6.27 to 110.94, p<0.0001) (table 2). Distribution of MABC-2 scores for different GA groups is illustrated in figure 2. No differences in background characteristics (neonatal and perinatal factors and sociodemographic factors) or comorbidity factors at 12 years were found in the control group with and without significant motor difficulties (online supplemental eTable 4).

**Figure 2:**
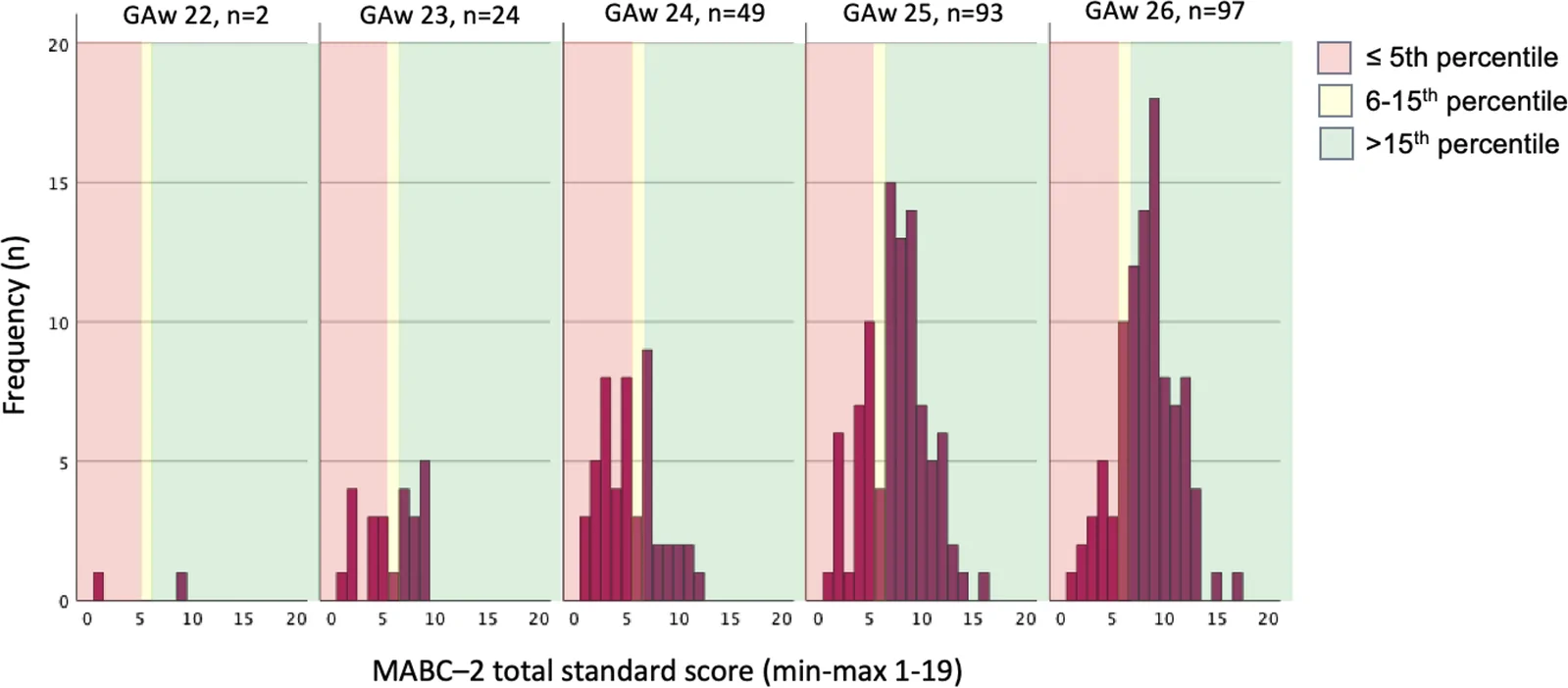
Frequency of MABC-2 total score (min–max 1–19) in children born extremely preterm by GAw. GAw, gestational age weeks; MABC-2, Movement Assessment Battery for Children-second edition.

**Table 2:** Prevalence of significant motor difficulties in children born extremely preterm (without CP and/or other severe NDI) and term-born controls at 12 years of age

Movement ABC-2 Score ≤5 percentile	Extremely preterm born n=268	Term-born controls n=196	Adjusted OR (95% CI)*	P value†
Manual dexterity, No. (%)	80 (30) n.d n=2	11 (6) n.d n=1	7.69 (3.88 to 15.25)	<0.0001
Ball skills (aiming/catching), No. (%)	91 (34)	15 (8)	6.27 (3.45 to 11.40)	<0.0001
Balance skills, No. (%)	51 (19) n.d n=1	2 (1)	26.38 (6.27 to 110.94)	<0.0001
Total score, No. (%)	79 (30) n.d n=3	5 (3) n.d n=1	16.99 (6.64 to 43.47)	<0.0001

* Adjusted for sex and multiple birth.

† Significant after Bonferroni-Holm adjustment.

CP, cerebral palsy; Movement ABC-2, Movement Assessment Battery for Children-second edition; n.d., no data; NDI, neurodevelopmental impairment.

In sensitivity analyses excluding children with moderate and severe cognitive impairment and adjusting for sex and multiple birth, children born EPT remained at substantially higher risk of significant motor difficulties across all MABC-2 domains, including manual dexterity, ball skills, balance and total score (online supplemental eTable 5).

An additional sensitivity analyses for the prevalence of significant motor difficulties comparing children born EPT versus term-born controls, restricted to only matched pairs with available MABC-2 total score was performed. The results were consistent with the main analyses, showing a significant difference between the two groups. Children born EPT had substantially higher odds of overall motor difficulties than term-born controls (aOR 20.89, 95% CI 6.18 to 70.65; p<0.0001) (online supplemental eTable 6).

### Motor difficulties and associated risk factors and comorbidities

Results from the univariable and multivariable regression models are reported in table 3. The AUC for the multivariable regression model was 0.707 and the number of observations included in the model was 264/268 (98.5%). Significant independent predictors for the prevalence of motor difficulties in EPT-born children were male sex (1.95, 95% CI 1.10 to 3.45, p=0.02) and GA (for every week decrease 2.02, 95% CI 1.52 to 2.69, p<0.0001).

**Table 3:** Univariable and multivariable analysis identifying predictors for the prevalence of significant motor difficulties at 12 years in children born extremely preterm (without CP and/or other severe NDI)

Factors	Missing	MABC-2 total score ≤5 percentile No./total No. (%)	Univariable model		Multivariable model	
			OR (95% CI)	P value	OR (95% CI)	P value
Sex						
Female	0	29/126 (23)				
Male	0	50/139 (36)	1.86 (1.08 to 3.19)	0.03	1.95 (1.10 to 3.45)	0.02
Gestational age, weeks (by 1 week decrease)						
22	0	1/2 (50)				
23	0	11/24 (46)				
24	0	28/49 (57)				
25	0	25/93 (27)				
26	0	14/97 (14)	1.98 (1.50 to 2.62)	<0.0001*	2.02 (1.52 to 2.69)	<0.0001
Birth weight (z-score) (by one unit decrease)						
≤median	0	44/134 (33)				
>median	0	35/131 (27)	0.87 (0.70 to 1.08)	0.20		
Apgar score at 5 min						
Score ≥7	0	49/181 (27)				
Score <7	0	30/84 (36)	1.49 (0.85 to 2.58)	0.16		
Pre-eclampsia						
No	13	64/218 (29)				
Yes	13	12/34 (35)	1.30 (0.61 to 2.79)	0.49		
Parity						
First	0	49/161 (30)				
Second	0	15/60 (25)	0.76 (0.38 to 1.48)	0.41		
Third	0	9/30 (30)	0.97 (0.41 to 2.27)	0.95		
Fourth	0	6/14 (43)	1.70 (0.56 to 5.16)	0.35		
Multiple birth						
Single	1	63/212 (30)				
Twin	1	14/45 (31)	1.07 (0.53 to 2.14)	0.85	1.02 (0.49 to 2.14)	0.96
Triplet	1	2/7 (29)	0.95 (0.18 to 5.01)	0.95	1.52 (0.28 to 8.37)	0.63
Antenatal steroids						
No	10	3/15 (20)				
Yes, incomplete	10	13/59 (22)	1.13 (0.28 to 4.62)	0.86		
Yes, complete	10	62/181 (34)	2.10 (0.57 to 7.73)	0.26		
Postnatal steroids						
No	0	55/208 (26)				
Yes	0	24/57 (42)	2.01 (1.09 to 3.70)	0.03		
Days on mechanical ventilation,						
0–7 days	0	27/135 (20)				
8–28 days	0	33/97 (34)	2.04 (1.13 to 3.71)	0.02		
>28 days	0	19/33 (58)	5.38 (2.39 to 12.08)	<0.0001*		
Birth delivery						
Vaginal birth	0	35/111 (32)				
Forceps delivery	0	0/1 (0)	0.00 (0.00 to 1)	0.99		
Caesarean section	0	44/153 (29)	0.88 (0.52 to 1.51)	0.65		
Intraventricular haemorrhage						
No	1	73/248 (29)				
Yes, grade 3–4	1	6/16 (38)	1.43 (0.50 to 4.08)	0.50		
Retinopathy of prematurity						
Stage II or less	2	47/187 (25)				
Stage III–IV	2	31/76 (41)	2.04 (1.16 to 3.58)	0.01		
Periventricular leukomalacia						
No	0	78/262 (30)				
Yes	0	1/3 (33)	1.17 (0.10 to 13.13)	0.90		
Necrotising enterocolitis						
No	0	73/251 (29)				
Yes, surgically treated	0	6/14 (43)	1.82 (0.61 to 5.43)	0.28		
Bronchopulmonary dysplasia†						
No	24	18/88 (21)				
Yes	24	51/153 (33)	1.96 (1.06 to 3.64)	0.03		
Patent ductus arteriosus						
No	0	56/205 (27)				
Yes, ligated	0	23/60 (38)	1.64 (0.90 to 3.01)	0.11		
Sepsis						
No	0	38/145 (27)				
Yes, verified	0	41/123 (33)	1.36 (0.80 to 2.30)	0.26		
Mother’s age at infant delivery, years (by 1 year increase)						
≤median	0	39/136 (29)				
>median	0	40/129 (31)	1.03 (0.98 to 1.08)	0.20		
Maternal educational level						
University/college	0	38/139 (27)				
<University/college	0	41/126 (33)	1.27 (0.75 to 2.15)	0.38		
Maternal country of birth						
Nordic	4	61/218 (28)				
Non-nordic	4	16/43 (37)	1.52 (0.76 to 3.01)	0.23		

The AUC for the multivariable model was 0.707 and the number of observations included in the model was 264/268 (98.5%).

The multivariable model was selected using stepwise selection method. The univariable analyses that were significant after Bonferroni-Holm adjustment were selected for the stepwise model. The variables sex, gestational age (weeks) and multiple birth were forced into the model at all stages of the selection process and were used as adjustment variables.

* Significant after Bonferroni-Holm adjustment.

† Bronchopulmonary dysplasia, supplementary oxygen at 36 weeks of gestational age

AUC, area under the curve; CP, cerebral palsy; MABC-2, Movement Assessment Battery for Children-second edition; NDI, neurodevelopmental impairment.

### Parental ratings of overall motor function and motor-related healthcare

30–37% of the parents of EPT-born children with significant motor difficulties according to MABC-2 reported that their child had gross and/or fine motor difficulties. This was a significantly higher number than parents of EPT-born children without difficulties, where 7–9% reported problems (p<0.0001). According to the parents, only 9.5% of all children with motor difficulties had seen a physiotherapist during the last year (online supplemental eTable 1).

## Discussion

The main finding of this study was a significantly higher prevalence of motor difficulties among EPT-born children without CP and/or other severe NDI compared with term-born children, with difficulties persisting into school age. Approximately 30% of the EPT-born children displayed significant motor difficulties at 12 years versus only 3% of term-born children, even after adjustments for confounders. These children were also more likely to have neurodevelopmental disorders like ASD and ADHD. The risk factors associated with motor difficulties in EPT-born children presented in this study included male sex and lower GA. Parental reports showed that only a third of parents of EPT-born children with motor difficulties recognised this issue, and only 10% reported that their child had received recent physiotherapy.

In recent years, research has primarily focused on early identification of motor difficulties in EPT-born children to be able to initiate early interventions. A continuous evaluation of these abilities during later ages is also important since motor challenges can persist and impact overall health and quality of life.^18 19^ Consistent with other studies, we found a significantly higher prevalence of motor difficulties in EPT-born children than in term-born children.^6^ In our study, nearly a third (30%) of the included EPT-born children showed significant motor difficulties at 12 years, similar to the numbers presented by Aubert *et al*^22^ at 5 years. In a recent review from Panceri *et al*,^6^ the pooled estimated prevalence in EPT-born children at 3–13 years of age was reported to be 26%. However, the authors also highlight the difficulty comparing studies because of the heterogeneity among them. Bolk *et al*^3^ assessed the same cohort as in this study at 6.5 years, applying stricter exclusion criteria that omitted children also with moderate cognitive impairments. This resulted in a prevalence of motor difficulties of 37%, which was further reduced to 12.5% when using UK norms. In our study (using UK norms) applying the same stricter exclusion criteria at 12 years lowered the prevalence from 30% to 23% (online supplemental eTable 5). These variations highlight methodological challenges, particularly when comparing studies that use different norms and criteria and underscore the importance of longitudinal studies to better understand non-CP motor difficulties.^4^ Our findings suggest that the prevalence of motor difficulties in the EXPRESS cohort seems to be slightly higher at 12 years compared with 6.5 years. Future research will explore motor difficulty progression in this cohort.

In our study, the risk for significant motor difficulties in EPT-born children was particularly high for the balance subscale with an aOR of 26.38 (95% CI 6.27 to 110.94). This finding is critical because balance enables children to engage effectively in physical activities that are essential for development. Deficiencies in these areas can lead to challenges, potentially resulting in decreased physical fitness, social participation and self-esteem.^35^

Previously, different neonatal and perinatal characteristics have been associated with motor difficulties in EPT-born children, such as male sex, low GA, severe brain lesions, BPD and postnatal corticosteroids.^7 21 22 36^ Similarly, we found that in our population the risk factors shown to be associated with significant motor difficulties were male sex and lower GA. Male sex and lower GA are well-known risk factors for poor neurodevelopmental outcomes in preterm populations. Males are more vulnerable due to slower in utero brain maturation and lower levels of neuroprotective oestrogens, increasing susceptibility to white matter injury.^37^ Each week of earlier delivery significantly raises the risk of motor difficulties, which may be related to an increased risk for intraventricular haemorrhage and PVL but also to critical periods of brain development and motor pathway formation.^38^

In previous studies, sociodemographic characteristics have been explored showing an association between, for example, low maternal age at delivery, lower parental educational level and non-European maternal country of birth and risk of motor difficulties.^21 22^ In contrast to this, our results showed similar sociodemographic characteristics for the EPT-born children with and without motor difficulties.

Motor difficulties in children can significantly impact cognitive development, as motor skills underpin processes like spatial awareness, attention and problem-solving. These challenges often co-occur with neurodevelopmental disorders such as ASD and ADHD, as seen in our cohort, where ASD and ADHD were significantly more prevalent among EPT-born children with motor difficulties. Shared neural disruptions may affect both motor coordination and socio-communicative skills.^39^ ADHD further accentuates motor challenges through deficits in attention, executive functioning and impulse control, linked to cortical and subcortical disruptions in preterm children.^40^ A deeper understanding of how motor difficulties at this age affect attention, problem-solving and academic skills is an avenue of future research.

Parental reports provide a functional perspective on motor difficulties but often underestimate their severity compared with structured assessments, as parental questionnaires may lack sensitivity.^3 41 42^ In our study, only 30% and 37% of parents reported gross or fine motor difficulties, respectively, compared with 49% at 6.5 years in the same cohort,^3^ despite an increased percentage of children with significant motor difficulties at 12 years based on test results. This discrepancy suggests that motor difficulties may remain clinically relevant yet insufficiently recognised in everyday settings, particularly in the absence of overt neurological impairment. Such difficulties may nevertheless affect school participation, physical activity and psychosocial well-being, underscoring their relevance for daily functioning. Strikingly, only 9.5% of children with significant motor difficulties (per MABC-2) had seen a physiotherapist in the past year. For comparison, in a large European cohort, 43% of children with significant motor difficulties received motor-related healthcare at age 5.^26^ These findings are concerning, as therapy and access to motor-related healthcare may improve not only motor skills but also quality of life. Motor difficulties identified in childhood may be responsive to physiotherapy-based interventions, particularly when recognised early; however, long-term outcomes are heterogeneous. This underscores the importance of systematic follow-up to identify children who may benefit from targeted support rather than relying solely on parental recognition.

### Strength and limitations

This study’s strengths include the long-term follow-up of a population-based cohort of EPT-born children and term-born controls, offering valuable insights into motor difficulties at 12 years, a less-explored period. Standardised assessments ensured reliable comparisons, with subscale scores providing nuanced insights into specific motor skills. By analysing risk factors, such as sex, GA and neonatal morbidities, the study enhances understanding of biological vulnerabilities in EPT-born children. The study also includes parental perspectives on motor function and motor-related healthcare utilisation which adds a real-world context, highlighting shortcomings in the care provided today. Although focused on a Swedish population, findings may be generalisable to other contexts due to shared risk factors. However, differences in healthcare systems and living conditions require further studies to confirm generalisability.

Key limitations include the use of a non-validated parental questionnaire to evaluate the use of motor-related healthcare, complicating interpretation of the low rate (9.5%) of children with motor difficulties receiving physiotherapy. This may reflect limited access or underreporting and requires further investigation. As only a small number of descriptive items from the questionnaire were used in this study, reliability indices (eg, internal consistency) were not applicable. The cross-sectional design provides a snapshot at age 12, but longitudinal data would better illustrate trajectories for motor development. Lastly, dropouts in the EPT-born group, more commonly having PVL (even though small numbers in each group), lower birth weight in the dropouts in the control group and non-Nordic mothers in both groups, may underestimate motor difficulties, as these factors are associated with increased risk of motor difficulties.^21^

## Conclusion

Children born EPT without CP and/or other severe NDI face a high risk of motor difficulties at 12 years compared with term-born controls and these difficulties are associated with several comorbidities. Motor difficulties are underestimated by parents and findings suggest that children with difficulties don’t receive sufficient support. Our study highlights the importance of performing repeated assessment of motor function for high-risk children, throughout childhood and beyond the present national follow-up programmes, into mid-school age, so that children in need of intervention are not missed. Future studies will determine if questionnaires like the revised Developmental Coordination Disorder Questionnaire (DCDQ-07)^43^ or the Movement Assessment Battery for Children-third edition Checklist^44^ can be used in settings where motor assessments are not possible. In evaluations, additional comorbidities need to be taken into consideration for a deeper understanding of these children’s motor function and its impact on everyday activities.

## Supplementary material

10.1136/archdischild-2025-329687 online supplemental file 1

## Data Availability

No data are available.

## References

[1] Cheong JL, Spittle AJ, Burnett AC (2020). Have outcomes following extremely preterm birth improved over time?. Semin Fetal Neonatal Med.

[2] Spittle AJ, Cameron K, Doyle LW (2018). Motor Impairment Trends in Extremely Preterm Children: 1991-2005. Pediatrics.

[3] Bolk J, Farooqi A, Hafström M (2018). Developmental Coordination Disorder and Its Association With Developmental Comorbidities at 6.5 Years in Apparently Healthy Children Born Extremely Preterm. JAMA Pediatr.

[4] Spittle AJ, Dewey D, Nguyen T-N-N (2021). Rates of Developmental Coordination Disorder in Children Born Very Preterm. J Pediatr.

[5] Setänen S, Lehtonen L, Parkkola R (2016). The motor profile of preterm infants at 11 y of age. Pediatr Res.

[6] Panceri C, Sbruzzi G, Zanella LW (2024). Developmental coordination disorder in preterm children: A systematic review and meta-analysis. Eur J Neurosci.

[7] Evensen KAI, Ustad T, Tikanmäki M (2020). Long-term motor outcomes of very preterm and/or very low birth weight individuals without cerebral palsy: A review of the current evidence. Semin Fetal Neonatal Med.

[8] Broström L, Vollmer B, Bolk J (2018). Minor neurological dysfunction and associations with motor function, general cognitive abilities, and behaviour in children born extremely preterm. Dev Med Child Neurol.

[9] Bruggink JLM, Einspieler C, Butcher PR (2008). The quality of the early motor repertoire in preterm infants predicts minor neurologic dysfunction at school age. J Pediatr.

[10] Bruggink JLM, Einspieler C, Butcher PR (2009). Quantitative aspects of the early motor repertoire in preterm infants: do they predict minor neurological dysfunction at school age?. Early Hum Dev.

[11] Cameron KL, FitzGerald TL, McGinley JL (2021). Motor outcomes of children born extremely preterm; from early childhood to adolescence. Semin Perinatol.

[12] Nosko D, Broström L, Bolk J (2023). Changes in prevalence of non-optimal neurological condition between 6.5 and 12 years in children born extremely preterm. Eur J Paediatr Neurol.

[13] Örtqvist M, Einspieler C, Ådén U (2022). Early prediction of neurodevelopmental outcomes at 12 years in children born extremely preterm. Pediatr Res.

[14] Iverson JM (2022). Developing language in a developing body, revisited: The cascading effects of motor development on the acquisition of language. Wiley Interdiscip Rev Cogn Sci.

[15] Iverson JM (2021). Developmental Variability and Developmental Cascades: Lessons from Motor and Language Development in Infancy. Curr Dir Psychol Sci.

[16] Harris S, Wilmut K, Rathbone C (2021). Anxiety, confidence and self-concept in adults with and without developmental coordination disorder. Res Dev Disabil.

[17] Spittle AJ, FitzGerald T, Mentiplay B (2018). Motor impairments in children: More than just the clumsy child. J Paediatr Child Health.

[18] Aubert AM, Costa R, Johnson S (2023). Developmental motor problems and health-related quality of life in 5-year-old children born extremely preterm: A European cohort study. Dev Med Child Neurol.

[19] Mancini VO, Licari MK, Alvares GA (2024). Psychosocial wellbeing, parental concerns, and familial impact of children with developmental coordination disorder. Res Dev Disabil.

[20] Husby IM, Stray KM-T, Olsen A (2016). Long-term follow-up of mental health, health-related quality of life and associations with motor skills in young adults born preterm with very low birth weight. Health Qual Life Outcomes.

[21] Bolk J, Källén K, Farooqi A (2023). Perinatal risk factors for developmental coordination disorder in children born extremely preterm. Acta Paediatr.

[22] Aubert AM, Costa R, Ådén U (2022). Movement Difficulties at Age Five Among Extremely Preterm Infants. Pediatrics.

[23] Dannemiller L, Mueller M, Leitner A (2020). Physical Therapy Management of Children With Developmental Coordination Disorder: An Evidence-Based Clinical Practice Guideline From the Academy of Pediatric Physical Therapy of the American Physical Therapy Association. Pediatr Phys Ther.

[24] Smits-Engelsman B, Vinçon S, Blank R (2018). Evaluating the evidence for motor-based interventions in developmental coordination disorder: A systematic review and meta-analysis. Res Dev Disabil.

[25] Blank R, Barnett AL, John Cairney I (2019). International clinical practice recommendations on the definition, diagnosis, assessment, intervention, and psychosocial aspects of developmental coordination disorder. Dev Med Child Neurol.

[26] Costa R, Aubert AM, Seppänen A-V (2022). Motor-related health care for 5-year-old children born extremely preterm with movement impairments. Dev Med Child Neurol.

[27] Serenius F, Källén K, Blennow M (2013). Neurodevelopmental outcome in extremely preterm infants at 2.5 years after active perinatal care in Sweden. JAMA.

[28] Serenius F, Ewald U, Farooqi A (2016). Neurodevelopmental Outcomes Among Extremely Preterm Infants 6.5 Years After Active Perinatal Care in Sweden. JAMA Pediatr.

[29] Fellman V, Hellström-Westas L, Norman M (2009). One-Year Survival of Extremely Preterm Infants After Active Perinatal Care in Sweden. JAMA.

[30] Wechsler D (2014). Wechsler Intelligence Scale for Children-Fifth Edition.

[31] Henderson SE, Barnett AL (2007). Movement Assessment Battery for Children - 2.

[32] www.socialstyrelsen.se/en/statistics-and-data/registers/national-patient-register.

[33] Ludvigsson JF, Andersson E, Ekbom A (2011). External review and validation of the Swedish national inpatient register. BMC Public Health.

[34] Idring S, Rai D, Dal H (2012). Autism spectrum disorders in the Stockholm Youth Cohort: design, prevalence and validity. PLoS One.

[35] Macdonald K, Milne N, Orr R (2020). Associations between motor proficiency and academic performance in mathematics and reading in year 1 school children: a cross-sectional study. BMC Pediatr.

[36] van Hoorn JF, Schoemaker MM, Stuive I (2021). Risk factors in early life for developmental coordination disorder: a scoping review. Dev Med Child Neurol.

[37] Hintz SR, Kendrick DE, Vohr BR (2006). Gender differences in neurodevelopmental outcomes among extremely preterm, extremely‐low‐birthweight infants. Acta Paediatr.

[38] de Kieviet JF, Piek JP, Aarnoudse-Moens CS (2009). Motor development in very preterm and very low-birth-weight children from birth to adolescence: a meta-analysis. JAMA.

[39] Johnson S, Marlow N (2011). Preterm birth and childhood psychiatric disorders. Pediatr Res.

[40] Bröring T, Oostrom KJ, van Dijk-Lokkart EM (2018). Attention deficit hyperactivity disorder and autism spectrum disorder symptoms in school-age children born very preterm. Res Dev Disabil.

[41] Dewey D, Creighton DE, Heath JA (2011). Assessment of developmental coordination disorder in children born with extremely low birth weights. Dev Neuropsychol.

[42] Ke L, Barnett AL, Wang Y (2021). Discrepancies between Parent and Teacher Reports of Motor Competence in 5-10-Year-Old Children with and without Suspected Developmental Coordination Disorder. Children (Basel).

[43] Wilson BN, Crawford SG, Green D (2009). Psychometric properties of the revised Developmental Coordination Disorder Questionnaire. Phys Occup Ther Pediatr.

[44] Henderson SE, Barnett AL, Sudgen DA (2022). Movement Assessment Battery for Children – Third Edition (Movement ABC-3).

[45] Marsál K, Persson PH, Larsen T (1996). Intrauterine growth curves based on ultrasonically estimated foetal weights. Acta Paediatr.

