# Supplementary material for: Motor difficulties and associated risk factors at 12 years in children born extremely preterm: a population-based cohort study
Source: Arch Dis Child. 2026 May 8;111(11):e329687. doi: 10.1136/archdischild-2025-329687 (PMC13691701; doi:10.1136/archdischild-2025-329687)
Supplement: Supplementary file 1 [file archdischild-111-11-s001.pdf]

SUPPLEMENTARY ON-LINE MATERIAL

1. **eTable 1.** Parental ratings (EXPRESS health questionnaire) of motor function and obtained physiotherapy at 12 years for children born extremely preterm (without CP and/or other severe NDI) with and without significant motor difficulties.

2. **eTable 2.** Background characteristics of eligible children born extremely preterm (without CP and/or other severe NDI) with and without Movement Assessment Battery for Children-2 data at 12 years

3. **eTable 3.** Background characteristics of eligible children born at term with and without Movement Assessment Battery for Children-2 data at 12 years

4. **eTable 4.** Descriptive characteristics and co-morbidity factors at 12 years for children born at term with and without significant motor difficulties according to the Movement Assessment Battery for Children – 2nd ed.

5. **eTable 5.** Adjusted analyses for the prevalence of significant motor difficulties comparing children born extremely preterm (without CP and/or other severe NDI) and children born at term, excluding children both with moderate and severe cognitive impairment (<2 SD on the Wechsler Intelligence Scale for Children – V ed)

6. **eTable 6** Matched analyses for the prevalence of significant motor difficulties comparing children born EPT vs. full-term born controls (complete matched pairs with an available total score)

eTable 1. Parental ratings (EXPRESS health questionnaire) of motor function and obtained physiotherapy at 12 years for children born extremely preterm (without CP and/or other severe NDI) with and without significant motor difficulties.

	**MABC-2 total score ≤ 5th percentile, n (%)** n=79	**MABC-2 total score > 5th percentile, n (%)** n=186	**p-value**
**Gross motor difficulties**[a]			<.0001

	MABC-2 total score ≤ 5th percentile, n (%) n=79	MABC-2 total score > 5th percentile, n (%) n=186	p-value
No	48 (63.2)	162 (89.5)	
Yes	23 (30.3)	16 (8.8)	
Don’t know	5 (6.6)	3 (1.7)	
Missing	3	5	
**Fine motor difficulties**[b]			<.0001
No	42 (55.3)	157 (88.7)	
Yes	28 (36.8)	13 (7.3)	
Don’t know	6 (7.9)	7 (4.0)	
Missing	3	9	
**Physiotherapy contact**[c] (last 12 months)			0.025
No	64 (86.5)	162 (91.5)	
Yes	7 (9.5)	15 (8.5)	
Don’t know	3 (4.1)	0	
Missing	5	9	

Items from the questionnaire: [a] Does the child have any gross motor difficulties? [b]Does the child have any fine motor difficulties? [c]Is the child in contact with a physiotherapist?

eTable 2. Background characteristics of eligible children born extremely preterm (without CP and/or other severe NDI) with and without Movement Assessment Battery for Children-2 data at 12 years

	**Extremely preterm born with MABC-2 data Included** n=268	**Extremely preterm born without MABC-2 data Not included** n=109	**p-value**	**Difference between included vs. not included (95% CI)**
**Perinatal and neonatal factors**				
Sex, boy, n (%)	139 (51.9)	60 (55.0)	0.65	-3.2% (-14.3%; 7.9%)
Gestational age, weeks, mean ± SD<br>Median (range)	25.0±1.0<br>25.0 (22.0 - 26.0)	25.0±1.0<br>25.0 (22.0 - 26.0)	0.65	-0.05 (-0.28; 0.17)
Birth weight, g, mean ± SD	792.9±164.5	793.2±179.7	0.99	-0.25 (-38.00; 37.50)
Birth weight, z-score, mean ± SD	-0.7±1.2	-0.8±1.3	0.62	0.07 (-0.20; 0.34)
Small for gestational age[a], n (%)	44 (16.4)	17 (15.6)	1.00	0.8% (-7.3%; 8.9%)
Apgar score at 5min <7, n (%)	84 (31.3)	37 (33.9)	0.63	-2.6% (-13.1%; 7.9%)
Pre-eclampsia, n (%)	35 (13.7)<br>n.d. n=13	8 (7.8)<br>n.d. n=7	0.15	5.9% (-0.8%; 12.6%)
Primiparity, n (%)	162 (60.4)	65 (59.6)	0.13	0.8% (-10.1%; 11.7%)
Multiple birth, n (%)	52 (19.4)<br>n.d. n=1	12 (11.0)	0.04	8.5% (0.9%; 16.0%)
Antenatal steroids (incomplete and complete), n (%)	243 (90.7)<br>n.d. n=10	99 (90.8)<br>n.d. n=6	0.74	-1.9% (-6.6%; 2.8%)
Postnatal steroids	58 (21.6)	34 (31.2)	0.06	-9.6% (-19.5%; 0.4%)
Days on mechanical ventilation, median (range)	7.0 (0.0 - 121.0)	8.0 (0.0 - 66.0)	0.28	-1.0 (-3.0; 1.0)*
0-7 days	136 (50.7)	50 (45.9)		4.9% (-6.2%; 16.0%)

	Extremely preterm born with MABC-2 data Included n=268	Extremely preterm born without MABC-2 data Not included n=109	p-value	Difference between included vs. not included (95% CI)
8-28 days	98 (36.6)	45 (41.3)	0.53	-4.7% (-15.6%; 6.2%)
>28 days	34 (12.7)	14 (12.8)		-0.2% (-7.6%; 7.3%)
Cesarean section	156 (58.2)	59 (54.1)	0.61	4.1% (-7.0%; 15.1%)
Intraventricular hemorrhage, grade 3-4, n (%)	17 (6.4) n.d. n=1	4 (3.7) n.d. n=2	0.46	2.6% (-2.0%; 7.3%)
Retinopathy of prematurity, stage III-IV, n (%)	77 (28.9) n.d. n=2	32 (29.9) n.d. n=2	0.90	-1.0% (-11.2%; 9.3%)
Periventricular leukomalacia, n (%)	3 (1.1)	6 (5.5)	0.02	-4.4% (-8.8%; 0.1%)
Necrotizing enterocolitis, surgically treated, n (%)	15 (5.6)	2 (1.8)	0.17	3.8% (0.0%; 7.5%)
Bronchopulmonary dysplasia[b], n (%)	155 (63.5) n.d. n=24	65 (67.0) n.d. n=12	0.62	-3.5% (-14.6%; 7.7%)
Patent ductus arteriosus, ligated, n (%)	61 (22.8)	32 (29.4)	0.19	-6.6% (-16.5%; 3.3%)
Sepsis, verified, n (%)	125 (46.6)	51 (46.8)	1.00	-0.1% (-11.3%; 11.0%)
**Socio-demographic factors**				
Mother's age at infant delivery (years)	31.2±5.8 31.0 (17.0 - 46.0)	31.0±6.2 32.0 (17.0 - 43.0)	0.78	0.19 (-1.13; 1.51)
Maternal educational level, university/college, n (%)	141 (52.6)	46 (42.6) n.d. n=1	0.09	10.0% (-1.1%; 21.1%)
Maternal country of birth, non-nordic, n (%)	44 (16.7) n.d n=4	28 (27.2) n.d. n=6	0.03	-10.5% (-20.2%; -0.8%)
**Follow-up characteristics at 12 years**				
Autism spectrum disorder, n (%)	29 (10.8)	16 (14.7)	0.30	-3.9% (-11.5%; 3.8%)

	Extremely preterm born with MABC-2 data Included n=268	Extremely preterm born without MABC-2 data Not included n=109	p-value	Difference between included vs. not included (95% CI)
Attention deficit hyperactivity disorder, n (%)	55 (20.5)	21 (19.3)	0.89	1.3% (-7.6%; 10.1%)
Language disorder, n (%)	19 (7.1)	15 (13.8)	0.05	-6.7% (-13.8%; 0.5%)
Intellectual disability (mild) n (%)	6 (2.2)	4 (3.7)	0.48	-1.4% (-5.4%; 2.5%)

Abbreviations: MABC-2, Movement Assessment Battery for Children 2nd edition
[a] Small for gestational age, birth weight z-score ≤ 2 SD, [b] Bronchopulmonary dysplasia, supplementary oxygen at 36 weeks of gestational age.
Data are presented as mean±standard deviation, median (range) and number of observations, or number (percentage).
For test between two groups with respect to dichotomous variables Fisher's exact test was used, for ordered categorical variables Mantel-Haenszel Chi-square trend test, Chi-square test for unordered categorical variables, and for continuous variables Mann-Whitney U test or two-sample t-test.
For categorical variables the difference between groups were calculated as difference in proportion with corresponding 95% CI. For continuous normally distributed variables, the mean difference between groups is presented with corresponding 95% CI. [*]For continuous non-normally distributed variables, Hodges-Lehmann estimator is presented, corresponding to the
median of all pairwise differences in the outcome between the groups, along with 95% confidence intervals.

eTable 3. Background characteristics of eligible children born at term with and without Movement Assessment Battery for Children-2 data at 12 years

	**Term-born children with MABC-2 data Included** n=196	**Term-born children without MABC-2 data Not included** n=176	**p-value**	**Difference between included vs. not included (95% CI)**
**Perinatal and neonatal factors**				
Sex, boy, n (%)	112 (57)	94 (53)	0.53	3.7% (-6.4%; 13.8%)
Gestational age, weeks, mean ± SD<br>Median (range)	39.5±1.1<br>40.0 (37.0 - 41.0)	39.5±1.1<br>40.0 (37.0 - 41.0)	0.89	-0.02 (-0.24; 0.21)
Birth weight, g, mean ± SD	3674.1±450.6	3551.7±508.1	0.02	119.46 (21.73; 217.19)
Birth weight, z-score, mean ± SD	0.3±0.9	-0.03±1.1	0.01	0.28 (0.08; 0.49)
Small for gestational age[a], n (%)	1 (1)	5 (3)	0.11	-2.3% (-5.0%; 0.3%)
Primiparity, n (%)	90 (46)	92 (52)	0.09	-6.4% (-16.5%; 3.8%)
Multiple birth, n (%)	0 (0)	0 (0)		
**Socio-demographic factors**				
Mother’s age at infant delivery (years)	31.2±4.8	31.3±4.4	0.90	-0.06 (-1.00; 0.88)
Maternal educational level, university/college, n (%)	140 (71)	109 (62)	0.05	10.1% (0.5%; 19.6%)
Maternal country of birth, non-nordic, n (%)	5 (3)	14 (8)	0.03	-5.4% (-10.0%; -0.8%)
**Follow-up characteristics at 12 years**				
Autism spectrum disorder, n (%)	2 (1)	4 (2)	0.26	-1.8% (-4.6%; 1.0%)
Attention deficit hyperactivity disorder, n (%)	16 (8)	17 (10)	0.59	-2.1% (-8.0%; 3.8%)

	Term-born children with MABC-2 data Included n=196	Term-born children without MABC-2 data Not included n=176	p-value	Difference between included vs. not included (95% CI)
Language disorder, n (%)	1 (1)	2 (1)	0.60	-0.6% (-2.5%; 1.2%)
Intellectual disability (mild) n (%)	0 (0)	0 (0)		

Abbreviations: MABC-2, Movement Assessment Battery for Children 2nd edition

[a] Small for gestational age, birth weight z-score ≤ 2 SD.

Data are presented as mean±standard deviation, median (range) and number of observations, or number (percentage). For test between two groups with respect to dichotomous variables Fisher's exact test was used, for ordered categorical variables Mantel-Haenszel Chi-square trend test, and for continuous variables two-sample t-test.

For categorical variables the difference between groups were calculated as difference in proportion with corresponding 95% CI.

For continuous normally distributed variables, the mean difference between groups is presented with corresponding 95% CI.

eTable 4. Descriptive characteristics and co-morbidity factors at 12 years for children born at term with and without significant motor difficulties according to the Movement Assessment Battery for Children – 2nd ed.

Characteristics	MABC-2 total score ≤ 5th percentile n=5	MABC-2 total score > 5th percentile n=190	p-value
**Perinatal and neonatal factors**			
Sex, boy, n (%)	4 (80.0)	107 (56.3)	0.39
Gestational age, weeks, mean ± SD	40.0±0.7	39.5±1.1	0.28
Birth weight, gram, mean ± SD	3558.0±578.5	3677.5±449.3	0.56
Birth weight, z-score, mean ± SD	-0.3±1.0	0.3±0.9	0.18
Small for gestational age, (birth weight z-score ≤ 2 SD), n (%)	0	1 (0.5)	1.00
Primiparity, n (%)	4 (80.0)	86 (45.3)	0.35
Multiple birth, n (%)	0	0	
**Socio-demographic factors**			
Mother's age at infant delivery, years, mean ± SD	28.2±5.8	31.3±4.8	0.15
Maternal educational level university/college, n (%)	4 (80.0)	136 (71.6)	1.00
Maternal country of birth, non-nordic, n (%)	1 (20.0)	4 (2.1)	0.12
**Follow-up characteristics at 12 years**			
Autism spectrum disorder, n (%)	0	2 (1.1)	1.00
Attention deficit hyperactivity disorder, n (%)	1 (20.0)	15 (7.9)	0.35
Language disorder	0	1 (0.5)	1.00
Intellectual disability, n (%)	0	0	

eTable 5. Adjusted analyses for the prevalence of significant motor difficulties comparing children born extremely preterm (without CP and/or other severe NDI) and children born at term, excluding children both with moderate and severe cognitive impairment (<2 SD on the Wechsler Intelligence Scale for Children – V ed)

Movement ABC-2<br>Score ≤ 5th percentile	Extremely preterm born	Term-born controls	OR (95 CI)	p-value*
Manual dexterity, n (%)	50 (24.2)<br>n.d n=3	10 (5.3)	5.89 (2.82 – 12.31)	<.0001
Ball skills (aiming/catching), n (%)	60 (28.8)<br>n.d. n=1	15 (7.9)	5.13 (2.75 - 9.56)	<.0001
Balance skills, n (%)	34 (16.4)<br>n.d. n=2	2 (1.0)	20.78 (4.85 – 89.09)	<.0001
Total score, n (%)	48 (23.3)<br>n.d. n=4	4 (2.1)	15.23 (5.29 – 43.85)	<.0001

Adjusted for Sex and Multiple birth. * Significant after Bonferroni-Holm adjustment.

eTable 6. Matched analyses for the prevalence of significant motor difficulties comparing children born EPT vs. full-term born controls (complete matched pairs with an available total score)

Outcome	**Extremely preterm born** n=126	**Term-born controls** n=126	OR (95% CI)	p-value
MABC-2 total score ≤ 5th percentile	42 (33.3%)	3 (2.4%)	20.50 (4.96 - 84.75)	<.0001

Conditional logistic regression was used.
